# Evolution of floral scent in relation to self-incompatibility and capacity for autonomous self-pollination in the perennial herb *Arabis alpina*

**DOI:** 10.1093/aob/mcab007

**Published:** 2021-02-08

**Authors:** Hampus Petrén, Per Toräng, Jon Ågren, Magne Friberg

**Affiliations:** 1 Department of Biology, Lund University, Sölvegatan 37, SE-223 62 Lund, Sweden; 2 Department of Ecology and Genetics, Evolutionary Biology Centre, Uppsala University, Norbyvägen 18D, SE-752 36 Uppsala, Sweden; 3 SLU Swedish Species Information Centre, Box 7007, SE-750 07 Uppsala, Sweden

**Keywords:** *Arabis alpina*, floral scent, intraspecific variation, mating system, self-incompatibility, selfing syndrome, volatile organic compound (VOC)

## Abstract

**Background and Aims:**

The transition from outcrossing to selfing is a frequent evolutionary shift in flowering plants and is predicted to result in reduced allocation to pollinator attraction if plants can self-pollinate autonomously. The evolution of selfing is associated with reduced visual floral signalling in many systems, but effects on floral scent have received less attention. We compared multiple populations of the arctic–alpine herb *Arabis alpina* (Brassicaceae), and asked whether the transition from self-incompatibility to self-compatibility has been associated with reduced visual and chemical floral signalling. We further examined whether floral signalling differ between self-compatible populations with low and high capacity for autonomous self-pollination, as would be expected if benefits of signalling decrease with reduced dependence on pollinators for pollen transfer.

**Methods:**

In a common garden we documented flower size and floral scent emission rate and composition in eight self-compatible and nine self-incompatible *A. alpina* populations. These included self-compatible Scandinavian populations with high capacity for autonomous self-pollination, self-compatible populations with low capacity for autonomous self-pollination from France and Spain, and self-incompatible populations from Italy and Greece.

**Key Results:**

The self-compatible populations produced smaller and less scented flowers than the self-incompatible populations. However, flower size and scent emission rate did not differ between self-compatible populations with high and low capacity for autonomous self-pollination. Floral scent composition differed between self-compatible and self-incompatible populations, but also varied substantially among populations within the two categories.

**Conclusions:**

Our study demonstrates extensive variation in floral scent among populations of a geographically widespread species. Contrary to expectation, floral signalling did not differ between self-compatible populations with high and low capacity for autonomous self-pollination, indicating that dependence on pollinator attraction can only partly explain variation in floral signalling. Additional variation may reflect adaptation to other aspects of local environments, genetic drift, or a combination of these processes.

## INTRODUCTION

Flowering plants are remarkably diverse, with floral traits, pollinators and mating systems playing central roles in the processes of diversification and speciation (Stebbins, 1957; Barrett, 2002; Kay *et al*., 2006; Kay and Sargent, 2009; Goldberg *et al.*, 2010). The breakdown of self-incompatibility and the evolution of selfing is a common evolutionary transition that affects the structuring of genetic variation, the response to selection, the evolution of floral signals and rewards, and reproductive morphology in hermaphroditic plants (Barrett, 2002, 2010; Wright *et al.*, 2013; Cutter, 2019). The evolutionary significance of such transitions is evident both because mating system shifts can be associated with speciation (Foxe *et al.*, 2009; Wright *et al.*, 2013) and because diversification rates might differ between self-incompatible and self-compatible lineages (Stebbins, 1957; Goldberg *et al.*, 2010).

Self-incompatible plants are largely outcrossing (some selfing is possible if self-incompatibility is leaky; Raduski *et al.*, 2012), while outcrossing rates may vary widely among self-compatible populations (Schemske and Lande, 1985; Goodwillie *et al.*, 2005; Whitehead *et al.*, 2018). In self-compatible plants, the degree of spatial and temporal separation of male and female function determines the capacity for autonomous self-pollination (Toräng *et al.*, 2017), whereas the realized rate of selfing will also be affected by the abundance, behaviour and morphology of local pollinators (Whitehead *et al.*, 2018). A transition from outcrossing to selfing is most often attributed to selection for reproductive assurance when mate and/or pollinator availability is limited (Darwin, 1876; Baker, 1955; Wright *et al.*, 2013). In addition, selfing provides a transmission advantage compared with outcrossing, but may often result in inbreeding depression (Charlesworth, 2006).

The shift from outcrossing to autonomous self-pollination is commonly associated with the evolution of the selfing syndrome, which includes reduced floral display, reduced herkogamy (stigma–anther separation) and dichogamy (temporal separation of male and female function) and a decrease in pollen-to-ovule ratio (Baker, 1955; Sicard and Lenhard, 2011). The evolution of the selfing syndrome is likely adaptive, because reduced allocation to floral display should decrease costs of floral signalling, whereas reduced herkogamy and dichogamy, as well as shifts in anther orientation, may increase the capacity for autonomous self-pollination (Sicard and Lenhard, 2011; Shimizu and Tsuchimatsu, 2015; Toräng *et al.*, 2017). The great majority of studies examining the selfing syndrome in plants have focused on the effects of transitions from self-incompatibility to self-compatibility and shifts in mating system (outcrossing rate) on visual floral signals (e.g. flower colour and size) and floral morphology (e.g. Busch, 2005; Goodwillie *et al.*, 2010; Carleial *et al.*, 2017). Floral chemical signalling (scent) is important for pollinator attraction (Raguso, 2008; Wright and Schiestl, 2009) and could be expected to vary in much the same way as visual floral signals do, but the association between floral scent evolution and transitions from self-incompatibility to self-compatibility and autonomous self-pollination is poorly resolved.

We are aware of only four previous studies that have investigated the association between floral scent and variation in self-incompatibility and mating system. First, Raguso *et al.* (2007) found a strong reduction in scent emission in the putatively autonomously selfing *Oenothera flava* ssp. *flava* compared with the self-compatible but putatively mixed-mating *O. flava* ssp. *taraxacoides*. Second, autonomously selfing populations of *Abronia umbellata* showed a 99 % decrease in scent emission, and potentially a different scent composition, compared with outcrossing self-incompatible populations (Doubleday *et al.*, 2013). Third, Sas *et al.* (2016) showed that the autonomously selfing species *Capsella rubella* had a much reduced scent emission, most notably in benzaldehyde, compared with the outcrossing self-incompatible *C. grandiflora*. Finally, the self-compatible *Phlox cuspidata* was characterized by a slight reduction in emission of three scent compounds, but did not differ in total scent emission rate compared with the closely related outcrossing self-incompatible *P. drummondii* (Majetic *et al.*, 2019). The authors proposed that the lack of a strong reduction in scent emission could be due to a continued reliance on pollinators for pollen transfer in *P. cuspidata*, perhaps in combination with a defensive function of some compounds. Taken together, these results suggest that the evolution of autonomous self-pollination, which decreases the need to attract insect pollinators, is often associated with reduced floral scent, but that the evolution of self-compatibility need not necessarily by itself result in selection for reduced scent emission.

The hypothesis that shifts in pollinator dependence rather than the evolution of self-compatibility *per se* drive the reduction of floral scent can be tested by examining species that include both self-incompatible populations and self-compatible populations that differ in capacity for autonomous self-pollination, and hence reliance on pollinator attraction for fertilization. Such an analysis should take into account that floral scent may differ substantially between populations of the same or closely related species in the absence of variation in self-incompatibility or mating system (Soler *et al.*, 2011; Parachnowitsch *et al.*, 2012; Friberg *et al.*, 2014, 2019; Gross *et al.*, 2016; Chapurlat *et al.*, 2018). This variation might be related to differences in pollinator or herbivore assemblages, abiotic environment, biochemical constraints, historical factors and/or genetic drift (Delle-Vedove *et al.*, 2017). Therefore, a full understanding of the role of self-incompatibility and evolution of autonomous self-pollination for intraspecific floral scent variation requires the study of multiple populations.

Here we performed a greenhouse common-garden experiment to examine the relationship between variation in self-incompatibility and capacity for autonomous self-pollination on the one hand, and visual display (flower size) and floral scent (emission rate and composition) on the other, in the arctic–alpine herb *Arabis alpina*. Throughout its distribution, this species includes both self-incompatible populations and self-compatible populations that vary in capacity for autonomous self-pollination and outcrossing rate, making it particularly well suited for studies of ecological and evolutionary consequences of such variation. Self-incompatible populations are found in the Italian Apuan Alps, central Italy and Greece (Ansell *et al.*, 2008; Tedder *et al.*, 2011; Laenen *et al.*, 2018), whereas self-compatible populations are found in the French, Swiss and northern Italian Alps, Spain and Scandinavia (Ansell *et al.*, 2008; Tedder *et al.*, 2011; Buehler *et al.*, 2012; Toräng *et al.*, 2017). A recent study of several self-compatible populations documented markedly higher capacity for autonomous self-pollination in Scandinavian compared with French and Spanish populations, and showed that this was associated with reduced herkogamy, more introrse anthers and lower outcrossing rates (Toräng *et al.*, 2017). It is not known whether self-compatibility has a single origin or several origins in this species, but the most parsimonious hypothesis would include one shift from self-incompatibility to self-compatibility, and that Scandinavian populations subsequently evolved autonomous self-pollination (Ehrich *et al.*, 2007; Toräng *et al.*, 2017; Laenen *et al.*, 2018).

We quantified variation in flower size and in emission rate and composition of floral scent among 17 populations of *A. alpina*, and examined whether visual and chemical floral signals are reduced in self-compatible compared with self-incompatible populations, or, alternatively, if floral signalling is lower only in the self-compatible populations with a high capacity for autonomous self-pollination, i.e. in populations that no longer depend on pollinators for pollen transfer.

## MATERIALS AND METHODS

### Study species and populations

*Arabis alpina* (Brassicaceae) is an arctic–alpine perennial herb widely distributed in the northern hemisphere, occurring in mountain regions in Europe, East Africa, Asia Minor and eastern North America (Koch *et al.*, 2006). It grows mostly in disturbed, rocky habitats, and plants are often found beside small streams, along scree slopes and on rock ledges. Flowers are primarily visited by various dipteran, hymenopteran and lepidopteran insects, with notably higher pollinator activity in populations in southern Europe compared with populations in Scandinavia (H. Petrén and P. Toräng, pers. obs.). Our greenhouse experiment included a total of 575 individuals representing 404 maternal families, grown from seeds collected in 2013–15 in 17 natural *A. alpina* populations from various mountain regions across Europe [7–48 (median = 21) maternal families sampled per population; Table 1]. This included nine self-incompatible populations from Italy and Greece, and eight self-compatible populations from France, Spain and Scandinavia (Table 1). The self-compatible populations were included in a previous study that demonstrated high capacity for autonomous self-pollination and low outcrossing rates in four Scandinavian populations, and low capacity for autonomous self-pollination and comparatively higher outcrossing rates in four French and Spanish populations (Toräng *et al.*, 2017; Table 1). For the self-incompatible populations, we performed controlled self- and cross-pollinations in a pollinator-free greenhouse on 2–13 plants per population. This confirmed results from previous studies (Ansell *et al.*, 2008; Tedder *et al.*, 2011; Toräng *et al.*, 2017; Laenen *et al.*, 2018), indicating that the Italian and Greek populations are self-incompatible (H. Petrén and M. Friberg, unpubl. res.).

**Table 1.** Name, region, coordinates, altitude, number of individuals and maternal seed families scored for scent and flower size, presence of self-incompatibility (SC, self-compatible; SI, self-incompatible), outcrossing rate and efficiency of autonomous self-pollination for the 17 *A. alpina* study populations. For outcrossing rate, – indicates absence of data. For efficiency of autonomous self-pollination, NA indicates that measuring efficiency of autonomous self-pollination is not applicable to self-incompatible populations

Population	Region	Latitude	Longitude	Altitude (m)	Individuals	Seed families	Self-compatible/self-incompatible	Outcrossing rate^a,b^	Efficiency of autonomous self-pollination^a^
S1	Northern Scandinavia	68°24′ N	18°19′ E	680	33	28	SC	0.0169	0.844
S2	Northern Scandinavia	68°21′ N	18°43′ E	780	28	28	SC	0.139	0.794
S4	Central Scandinavia	62°50′ N	11°44′ E	960	39	36	SC	0.0134	0.782
S5	Central Scandinavia	63°12′ N	12°19′ E	1330	22	22	SC	0.0972	0.711
E3	Northwest Spain	43°14′ N	05°56′ W	1170	43	36	SC	0.231	0.378
E4	Northwest Spain	43°03′ N	06°06′ W	1760	35	31	SC	0.297	0.390
Fr1	French Alps	45°03′ N	06°24′ E	2190	50	48	SC	0.266	0.173
Fr2	French Alps	44°57′N	06°36′ E	1980	24	23	SC	0.139	0.427
It2	Central Italy	42°30′ N	13°35′ E	1660	36	19	SI	–	NA
It4	Central Italy	42°15′ N	13°19′ E	1050	31	16	SI	–	NA
It5	Central Italy	41°58′ N	13°33′ E	700	33	20	SI	–	NA
It6	Central Italy	41°50′ N	13°56′ E	1650	39	20	SI	–	NA
It7	Central Italy	41°55′ N	13°53′ E	950	35	19	SI	–	NA
It8	Northern Italy	44°08′ N	10°15′ E	900	37	20	SI	–	NA
It9	Northern Italy	44°05′ N	10°19′ E	910	38	21	SI	–	NA
G1	Northwest Greece	39°52′ N	20°46′ E	760	17	7	SI	–	NA
G2	Northwest Greece	39°57′ N	20°48′ E	1880	35	10	SI	–	NA

^a^Estimates from Toräng *et al.*, 2017.

^b^Estimated from multilocus structure of established plants.

### Experimental setup

Seeds were grown under common greenhouse conditions at Uppsala University in three cohorts in 2014, 2016 and 2017. We planted five seeds from each of 7–48 maternal plants per population in 6 × 6 × 7 cm pots filled with a soil mixture containing two-thirds potting soil (Yrkes-Plantjord, SW Horto, Hammenhög, Sweden) and one-third 2–6 mm LECA (Lightweight Expanded Clay Aggregate; Saint-Gobain Byggprodukter, Sollentuna, Sweden), with a top layer of sowing soil (Plugg och Såjord, SW Horto, Hammenhög, Sweden). The pots were stratified in dark conditions at 4 °C for 1 week before being transferred to a growth room maintaining a 16-h day with a temperature of 20 °C and light intensity of 150 µEm^−2^ s^−1^, and an 8-h, 16 °C night. Seeds from the two Greek populations sampled in 2017 were sown on agar plates, but otherwise treated in the same way. Following germination, normally one or two seedlings from each maternal plant family were transferred to individual pots filled with the same soil mix as above. After a total of 6 weeks of vegetative growth, plants were vernalized for 12 weeks in conditions with an 8-h, 4–6 °C, 50 µEm^−2^ s^−1^ day and a 16-h, 4–6 °C night. Following vernalization, plants were transferred to a greenhouse with both natural and artificial lighting, and a 16-h, 18 °C day and an 8-h, 16 °C night, which induced flowering. Plants were watered two or three times a week with water containing a low concentration of nutrients (SW Bouyant Rika T 3-1-5 fertilizer, SW Horto, Hammenhög, Sweden) and began flowering within 2 weeks of being transferred to the greenhouse.

### Flower size

A total of 575 plants were included for measurement of flower size and scent emission (Table 1). We determined the size of one newly opened flower per plant. Flower buds that were about to open were marked in the afternoon and measured the following day to ensure that flowers were measured at a similar age. The flower was removed from the plant and photographed from above using a Leica DFC450 microscope camera mounted on a Leica MZ8 stereomicroscope (Leica Microsystems, USA). Each flower was photographed with a millimetre paper for scale and images were analysed using the software ImageJ (Schneider *et al.*, 2012). For each flower, we recorded the flower diameter as the distance between the outer edges of two opposing petals. Flower size was recorded for individuals one to several days after they had been sampled for scent, to avoid potential effects of the flower removal on the emission rate or composition of the floral scent.

### Scent collection and analysis

Floral scent was collected using dynamic headspace sampling (Raguso and Pellmyr, 1998). For each sampled plant, we counted the number of open flowers (mean = 10.7, s.d. = 4.3) in the inflorescence, which was then enclosed in a 12 × 14 cm oven bag (ICA Sverige, Solna, Sweden) with a small hole on top and larger opening at the bottom. We tried to collect scent at peak flowering, i.e. when as many flowers as possible were open for each individual. A Teflon tube scent trap with a 10-mg Tenax GR filter (Sigma–Aldrich, St Louis, MO, USA) was then attached to the bottom opening of the oven bag, and using a custom-built pump (GroTech, Gothenburg, Sweden) the air surrounding the enclosed inflorescence was pulled through the scent trap at a rate of 200 mL min^−1^ for a total of 3 h of volatile collection. The flow rate over each trap was kept constant using a Cole-Parmer 65-mm direct-reading flow meter (Vernon Hills, IL, USA). Sampling was started between 0900 and 1300 h, as *A. alpina* plants are most scented during daytime (H. Petrén and M. Friberg, unpubl. res.). At each sampling occasion, a control sample of ambient air drawn through an oven bag was collected the same way as described above. After sampling, we eluted the volatiles from the scent traps using 300 µL of ultrapure gas chromatography/mass spectrometry (GC/MS)-quality hexane and stored samples at −18 °C in glass vials. Before analysis, samples were concentrated to 50 µL using a moderate flow of nitrogen gas. Thereafter, an internal standard of 5 µL of 0.03 % toluene was added to each sample, to enable an estimation of the standardized emission rate per flower (Svensson *et al.*, 2005; Friberg *et al.*, 2013).

Scent samples were analysed using GC/MS on a Finnigan TRACE GC Ultra 2000 gas chromatograph connected to a Finnigan Trace DSQ mass spectrometer (ThermoFisher, Waltham, MA, USA). The gas chromatograph was equipped with a 30 m × 0.250 mm × 0.25 μm DB-Wax column (Agilent Technologies, Santa Clara, CA, USA) and used ultra-high purity (99.999 %) helium as a carrier gas at a constant velocity of 1 mL min^−1^. The temperature programme was initiated at 50 °C for 3 min before a temperature increase of 10 °C min^−1^ for 20 min, reaching a maximum temperature of 250 °C, where it was held constant for 7 min until the end of the programme. Peaks of floral volatiles in resulting chromatograms were manually integrated using the mass spectrometer manufacturer’s software (Xcalibur version 1.4, Thermo Electron Corporation 1998–2003, San José, CA, USA). Compounds were identified using MS library suggestions (NIST, version 2.0, 2008) and confirmed by comparisons with Kovats retention indices obtained from the literature, and for some compounds also through co-chromatography with synthetic standards (Supplementary Data Table S1). We excluded from further analysis compounds that were known to be green-leaf volatiles and compounds that had an emission rate <3-fold that of the corresponding control sample, as these were deemed to not be part of the actual floral scent emitted. Finally, for each sampled plant the emission rate of each compound and the total emission rate of all compounds was estimated in nanograms per hour per flower (Svensson *et al.*, 2005).

### Statistical analyses

We used a suite of statistical analyses to examine variation in flower size and floral scent among populations of the three mating system categories: self-incompatible populations (Greek and Italian), self-compatible populations with high capacity for autonomous self-pollination (Scandinavian), and self-compatible populations with low capacity for autonomous self-pollination (French and Spanish; Table 1). Analyses were conducted in R 3.5.2 (R Core Team, 2018) using the lme4 (Bates *et al.*, 2015), lmerTest (Kuznetsova *et al.*, 2017), multcomp (Hothorn *et al.*, 2008), randomForest (Liaw and Wiener, 2002) and vegan (Oksanen *et al.*, 2018) packages.

To investigate differences in floral signalling among the three mating system categories, we constructed linear mixed models with flower diameter and total scent emission rate per flower (log-transformed to meet test assumptions) for each plant as the response variables, mating system category as a fixed factor, and population nested within mating system category as a random factor. *Post hoc* pairwise comparisons of mating system categories were done using Tukey’s honest significant differences test (*glht* function in multcomp package).

To examine variation in the composition of the floral scent, we performed a set of multivariate statistical analyses on the relative proportions of scent compounds for each sampled plant. First, we visualized variation in scent composition by calculating Bray–Curtis dissimilarities between samples and illustrating them in a non-metric multidimensional scaling (NMDS) plot (*metaMDS* function in the vegan package). In a next step, we calculated average Bray–Curtis dissimilarities for all population pairs, and used these dissimilarity averages in a hierarchical cluster analysis [unweighted pair-group method based on arithmetic mean (UPGMA)] to visualize relations in scent composition between populations.

Second, we performed permutational multivariate analysis of variance (PERMANOVA; Anderson, 2001), using Bray–Curtis dissimilarities. This was done with mating system category as factor, using the population means of the relative proportions of scent compounds in each sample, and additionally using the individual samples with population as factor. These analyses were done to test if the floral scent composition differed significantly between mating system categories and/or populations, and to estimate how much variation was explained by these factors (*adonis* function in the vegan package, 9999 permutations). We also performed PERMANOVAs with population as factor separately in the three mating system categories to examine whether scent composition varied among populations also within these groups. Heterogeneous dispersion and unbalanced data may result in unreliable rejection rates in PERMANOVA (Anderson and Walsh, 2013). This could potentially affect the analysis of the whole data set (but see the Results section), but should be much less of an issue in subsequent PERMANOVAs within each category as differences in sample size (Table 1) and dispersion were generally smaller between populations in those analyses.

Third, we used the Random Forest classification algorithm (Breiman, 2001; Ranganathan and Borges, 2010) to examine classifications of populations into different groups and investigate the importance of different scent compounds for that classification. Random Forest is a machine-learning algorithm well suited for multivariate data and provides a measure of the relative importance of different variables for the classification. The analysis was done on the population means of the relative proportions of scent compounds in each sample, rather than individual samples, because of unequal sample sizes among populations. We used the algorithm to estimate the ‘out of bag’ probability of membership of different groups. This was done first for only two groups, with populations classified as either self-incompatible or self-compatible, and second for the same three mating system categories as used above, where the self-compatible populations were divided into those with high and low capacity for autonomous self-pollination (*randomForest* function in the randomForest package, *n*_tree_ = 10 000, *m*_try_ = 5). Additionally, we calculated the mean decrease in accuracy for the different variables in the model (for the model with self-incompatible and self-compatible groups only, as this provided a better classification; see Results section). This provides the relative importances of different scent compounds for the classification. Finally, we performed the same analyses as for flower diameter and total scent emission rate on benzaldehyde emission rate (square-root-transformed to meet test assumptions), as this compound was most important for the classification (see Results section).

Fourth, we tested for the presence of spatial autocorrelation to examine whether geographical distribution could explain variation in floral scent and flower size among self-compatible and self-incompatible populations, respectively. The two categories of populations were tested separately since their distributions do not overlap. For flower size and total scent emission rate, we tested for spatial autocorrelation of population mean values using Moran’s *I* (Moran, 1950). This test statistic ranges from −1 to 1, with statistically significant positive values indicating that similar values cluster together, and statistically significant negative values indicating that dissimilar values cluster together. For scent composition, we performed a Mantel test, where we tested for a correlation between the geographical distance between populations and their difference in scent composition (using the average Bray–Curtis dissimilarities for all population pairs).

In most populations, data were collected from more than one plant from some maternal families (Table 1). To determine whether this was likely to affect results, we re-ran the analyses described above using a data set consisting of one randomly chosen plant per family, which reduced total sample size from 575 to 404 individuals. Results for the reduced data set were fully consistent with those based on the full data set and only the latter are reported below.

## RESULTS

### Flower size

**Fig. 1.**
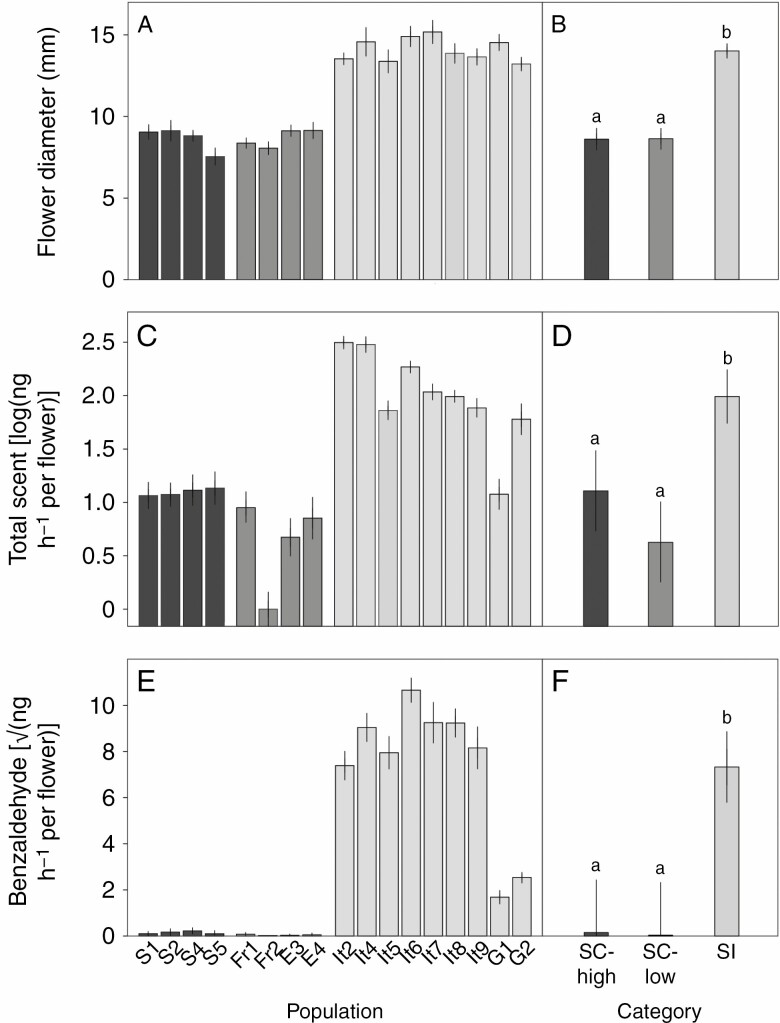
(A, B) Flower diameter, (C, D) total floral scent emission rate and (E, F) benzaldehyde emission rate of plants from the 17 *A. alpina* populations. (A, C, E) Means and 95 % confidence intervals for each population. (B, D, F) Means and 95 % confidence intervals (adjusted for population effects) for three categories of population: self-compatible with high capacity for autonomous self-pollination (SC-high); self-compatible with low capacity for autonomous self-pollination (SC-low); and self-incompatible (SI). Scandinavian populations (S1, S2, S4, S5; dark grey bars) are self-compatible with a high capacity for autonomous self-pollination. French and Spanish populations (Fr1, Fr2, French populations; E3, E4, Spanish populations; medium grey bars) are self-compatible with a low capacity for autonomous self-pollination. Greek and Italian populations (G1, G2, Greek populations; It2, It4, It5, It6, It7, It8, It9, Italian populations; light grey bars) are self-incompatible. See Table 1 for further information about populations. Different letters above bars in (B), (D) and (F) indicate significant differences between mating system categories (Tukey’s honest significant difference test).

Flower diameter varied significantly among the three mating system categories (*F*_2,13.42_ = 135.6, *P* < 0.001; Fig. 1B), with self-incompatible populations producing larger flowers than self-compatible populations. However, flower size did not differ between the groups of self-compatible populations with high and low capacity for autonomous self-pollination (Fig. 1B). Variation in flower diameter among populations within mating system categories was limited, but statistically significant (likelihood-ratio test (LRT), *χ*^2^_1_ = 47.35, *P* < 0.001; Fig. 1A).

### Floral scent

**Fig. 2.**
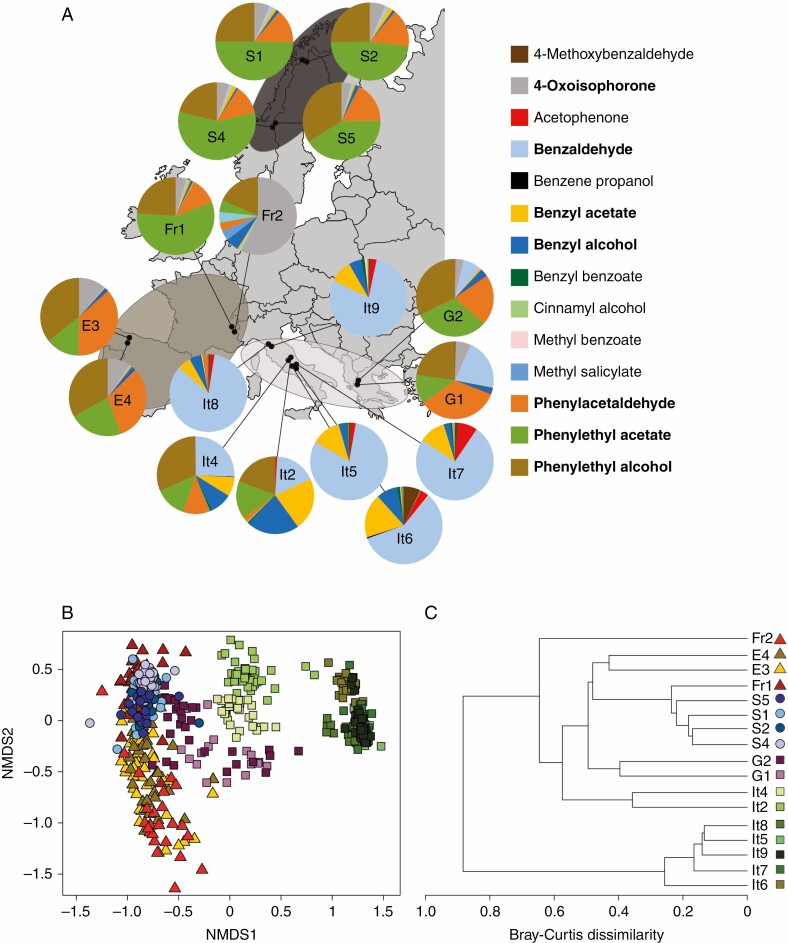
Visualization of floral scent variation among 17 European populations of *A. alpina*. (A) Map showing the location of each population, with pie charts indicating the population mean relative contribution of different scent compounds in the floral scent bouquet. Population names are the same as in Fig. 1. The 14 most common compounds are shown in the key, with the 7 compounds occurring in at least half of all samples in bold. Ellipses indicate distributions of self-incompatible populations (light grey) and self-compatible populations with a low (medium grey) and high (dark grey) capacity for autonomous self-pollination. (B) NMDS plot showing the distribution of floral scent samples from the 17 populations (2-D stress = 0.10). See panel (C) for explanation of symbols. Greek (purple) and Italian (green) populations are self-incompatible (squares). Scandinavian (blue) populations are self-compatible, with a high capacity for autonomous self-pollination (circles). French (red) and Spanish (yellow) populations are self-compatible, with a low capacity for autonomous self-pollination (triangles). Many data points are overlapping, most notably those of the Scandinavian populations in the top left and the Italian populations It5, It7, It8 and It9 in the rightmost cluster. Supplementary Data Fig. S2 shows the centroids for each population for a clearer visualization. (C) Clustering cladogram illustrating patterns of variation in floral scent composition among populations, based on average Bray–Curtis dissimilarities between populations.

In total, we detected 32 different floral scent compounds (28 aromatics and 4 terpenes) in the *A. alpina* samples (Supplementary Data Table S1). Seven compounds (4-oxoisophorone, benzaldehyde, benzyl acetate, benzyl alcohol, phenylacetaldehyde, phenylethyl acetate and phenylethyl alcohol) were present in at least half of all sampled plants and four of these (benzyl acetate, benzyl alcohol, phenylethyl acetate and phenylethyl alcohol) were present in all populations (Fig. 2A).

Total floral scent emission rate per flower varied significantly among the three mating system categories (*F*_2,13.71_ = 20.50, *P* < 0.001; Fig. 1D), with self-incompatible populations overall having a higher total emission rate than self-compatible populations. In contrast, emission rate did not differ significantly between the groups of self-compatible populations with high and low capacity for autonomous self-pollination (Fig. 1D). Additionally, emission rate varied among different populations within the mating system categories (LRT, *χ*^2^_1_ = 239.9, *P* < 0.001). Compared with variation in flower size, variation among populations within mating system categories was larger, with one self-incompatible population (G1) having an emission rate similar to the self-compatible populations, and one self-compatible population (Fr2) emitting almost no scent at all (Fig. 1C).

There was extensive variation in scent composition among populations (Fig. 2A). In the NMDS plot (Fig. 2B; Supplementary Data Fig. S1), samples from the self-incompatible Italian populations formed a distinct group, with a clear separation of two populations emitting smaller proportions of benzaldehyde (It2 and It4). Likewise, samples from the Scandinavian populations with high capacity for autonomous self-pollination grouped closely together. Samples from the French and Spanish populations varied more, partly overlapping with each other and the Scandinavian samples. The scent composition of samples from the two self-incompatible Greek populations was intermediate to that of populations from the other geographical regions. The hierarchical cluster analysis showed a similar pattern (Fig. 2C). The Italian populations clustered together in a separate branch, except for the two populations with the lowest proportion of benzaldehyde in their scent composition, which instead clustered with the other populations. The Greek, Spanish and Scandinavian populations clustered with the other populations from the same region, while one of the French populations (Fr1) had a floral scent composition more similar to that of the Scandinavian populations.

Results from the PERMANOVA analysis confirmed that the floral scent composition varied significantly among mating system categories (PERMANOVA, *F*_2,14_ = 5.13, *P* = 0.006) and populations (PERMANOVA, *F*_16,558_ = 179.8, *P* < 0.001), with especially the population factor explaining a substantial part of the variation in floral scent composition (population, *R*^2^ = 0.84; mating system category, *R*^2^ = 0.42). PERMANOVAs conducted separately for the different mating system categories revealed significant differences among self-incompatible populations (PERMANOVA, *F*_8,292_ = 166.9, *P* < 0.001) and among self-compatible populations with high (PERMANOVA, *F*_3,118_ = 12.21, *P* < 0.001) and low (PERMANOVA, *F*_3,148_ = 33.16, *P* < 0.001) capacity for autonomous self-pollination. Population explained a considerably larger proportion of the variation in scent composition among self-incompatible populations (*R*^2^ = 0.82) than among self-compatible populations with high (*R*^2^ = 0.24) and low (*R*^2^ = 0.40) capacity for autonomous self-pollination.

For the Random Forest analysis, the model with two groups, self-compatible and self-incompatible, classified 16 of the 17 populations correctly, with a mean out-of-bag probability of membership of the correct group of 83.1 % (Supplementary Data Fig. S2A). The model with three mating system categories, where the self-compatible group was divided into populations with high and low capacity for autonomous self-pollination, classified 15 of the 17 populations correctly, with a mean out-of-bag probability of membership of the correct group of 67.8 % (Supplementary Data Fig. S2B). Furthermore, the measures of variable importance for the first model indicated that benzaldehyde was the most important compound for the classification of samples as either self-incompatible or self-compatible (Table 2). Benzaldehyde emission varied significantly among the three mating system categories (*F*_2,13.91_ = 20.23, *P* < 0.001; Fig. 1F), and among populations within the mating system categories (LRT, *χ*^2^_1_ = 536.3, *P* < 0.001), with higher benzaldehyde emission rates in self-incompatible compared with self-compatible populations. All plants in the self-incompatible populations, but very few in the self-compatible populations, emitted benzaldehyde at detectable levels (Fig. 1E; Supplementary Data Fig. S3).

**Table 2.** The ten most important compounds, measured by the mean decrease in accuracy, for the classification of *A. alpina* populations as self-compatible or self-incompatible according to the Random Forest analysis

Compound	Mean decrease in accuracy
Benzaldehyde	0.086
4-Methoxybenzaldehyde	0.068
Benzyl alcohol	0.032
Benzyl acetate	0.020
4-Oxoisophorone	0.019
Benzyl benzoate	0.017
Methyl salicylate	0.017
Phenylethyl acetate	0.0094
Phenylethyl alcohol	0.0087
Acetophenone	0.0076

The Moran’s *I* tests indicated that there was no statistically significant spatial autocorrelation for flower size or total scent emission rate among self-compatible populations (flower size, *I* = 0.27, *P* = 0.32; total scent, *I* = 0.05, *P* = 0.49) or among self-incompatible populations (flower size, *I* = −0.10, *P* = 0.94; total scent, *I* = 0.26, *P* = 0.20). For scent composition, Mantel tests indicated positive but weak correlations between geographical distance and scent composition dissimilarity among self-incompatible (*r* = 0.51, *P* = 0.02) and among self-compatible populations (*r* = 0.30, *P* = 0.05). In both categories, neighbouring populations could have both highly similar and quite different floral scent composition (Supplementary Data Fig. S4).

## DISCUSSION

This study has documented considerable variation in floral visual display and scent among European populations of the arctic–alpine perennial herb *A. alpina*. Flowers were smaller and floral scent emission rates substantially lower in self-compatible compared with self-incompatible populations. However, contrary to predictions, these traits were not further reduced in the Scandinavian self-compatible populations with high capacity for autonomous self-pollination compared with the French and Spanish self-compatible populations with low capacity for autonomous self-pollination. Moreover, floral scent composition varied considerably both among and within the three mating system categories. As scent was collected from plants growing in a common environment, the results indicate that among-population differences in scent emission rate and composition have a genetic basis.

### Variation in floral scent emission rate and flower size

The self-compatible *A. alpina* populations emitted floral scent at a lower rate and produced smaller flowers compared with the self-incompatible populations (Fig. 1A–D). The difference in flower size between self-compatible and self-incompatible *A. alpina* populations is consistent with a geographically limited comparison between self-compatible and self-incompatible populations of this species in northern Italy and southeast France (Tedder *et al.*, 2015). Scent emission rates varied substantially among populations with similarly sized flowers (Fig. 1A, C), demonstrating that variation in floral scent is not simply a function of differences in flower size.

The difference in total emission rate per flower between self-incompatible and self-compatible populations of *A. alpina* is in concordance with two of the three previous studies of other taxa comparing scent emission of self-incompatible and self-compatible species (Sas *et al.*, 2016) or populations (Doubleday *et al.*, 2013), but in contrast to the similar total emission rates of self-compatible *P. cuspidata* and self-incompatible *P. drummondii* (Majetic *et al.*, 2019). The high floral scent emission rate and large flowers of self-incompatible Greek and Italian populations likely reflect their obligate reliance on pollinators for pollen transfer (Goodwillie *et al.*, 2010). For the French and Spanish self-compatible populations with a low capacity for autonomous self-pollination, one explanation for their lower emission rates and smaller flowers compared with the self-incompatible populations could be that high pollinator abundance and the resulting low pollen limitation has reduced the importance of strong floral signals for successful reproduction. This would allow other selective forces or genetic drift to decrease floral scent emission, thereby reducing potential ecological and metabolic costs of scent production (Wright and Schiestl, 2009; Junker and Blüthgen, 2010).

Surprisingly, the Scandinavian populations, which due to low pollinator activity and high capacity for autonomous self-pollination (Table 1; Toräng *et al.*, 2017) likely rely much less on insect pollinators for fertilization, did not show any further reduction in either flower size or floral scent emission compared with the French and Spanish self-compatible populations with low capacity for autonomous self-pollination (Fig. 1A–D). This result contrasts with the strong reduction in floral scent emission rate in the autonomously self-pollinating *O. flava* ssp. *flava* compared with the self-compatible but mixed mating *O. flava* ssp. *taraxacoides* (Raguso *et al.*, 2007). Instead, among-population variation in floral scent emission rate was larger in Spain and France, with one French population (Fr2) emitting very low amounts of floral scent. The lack of further reduction in signalling traits in the Scandinavian *A. alpina* populations with high capacity for autonomous self-pollination may be related to their recent divergence during a postglacial colonization bottleneck. This late split has led to limited genetic diversity in Scandinavian populations (Ehrich *et al.*, 2007; Laenen *et al.*, 2018) that could, possibly in combination with pleiotropic effects on other floral traits (Ashman and Majetic, 2006), have prevented further reductions of scent emission. Taken together, these results suggest that the degree to which plants depend on pollinators for pollen transfer cannot alone explain the variation in floral scent emission rate among populations of *A. alpina*.

Because self-compatible and self-incompatible populations are geographically separated, with self-compatible populations in western and northern Europe and self-incompatible populations in the southeast, it is not possible to separate conclusively the effect of reproductive system from possible effects of geographical structure and population history on flower size and emission rate. However, the differences in these traits are according to predictions based on a reduced need for pollinator attraction in self-compatible populations. Moreover, the lack of spatial autocorrelation for these traits within each mating system category suggests that geographical and population genetic structure do not strongly constrain their evolution.

### Floral scent composition differences between self-compatible and self-incompatible populations

The floral scent composition differed most notably between self-incompatible and self-compatible populations, demonstrating that the transition from self-incompatibility to self-compatibility has been associated with differential reduction in emission rates of different scent compounds. By contrast, differences in scent composition were comparatively smaller between self-compatible populations with a high and low capacity for autonomous self-pollination (Fig. 2B, C). One key difference between self-incompatible and self-compatible populations was the high emission of benzaldehyde in both absolute and proportional terms in the former group (Fig. 1E, F; Supplementary Data Fig. S3). The Random Forest analysis also indicated that benzaldehyde was the most important compound for the classification of samples as stemming from self-incompatible or self-compatible populations (Table 2). The role of benzaldehyde in floral scent has been demonstrated in two recent studies. Amrad *et al.* (2016) showed that benzaldehyde emission was lost in the transition from hawkmoth pollination in *Petunia axillaris* to hummingbird pollination in *P. exserta*. Similarly, Sas *et al.* (2016) demonstrated that benzaldehyde constituted a major compound of the floral scent in sampled accessions of the self-incompatible *C. grandiflora*, but was absent in the accessions sampled of the selfing *C. rubella*. Interestingly, in both these cases reduced benzaldehyde emission was associated with deactivation of orthologous *CNL* genes, which encode cinnamate-CoA ligase, a protein that catalyses the biosynthesis of benzenoids, including benzaldehyde. Benzaldehyde is an attractant to both pollinating and florivorous insects (Theis, 2006), and the direction of selection on benzaldehyde emission may have changed following shifts away from insect pollination in the hummingbird-pollinated *P. exserta* and the selfing *C. rubella*. Inactivation of the *CNL* genes is thought to have limited pleiotropic effects on the biosynthesis of other benzenoids (Amrad *et al.*, 2016; Sas *et al.*, 2016). This should reduce the likelihood that pleiotropy constrains any response to selection on this trait, and may have been conducive to the reduction in emission of this particular compound. *Capsella* and *Arabis* are both members of the Brassicaceae, and it would be interesting to determine whether the same genetic mechanism explains the loss of benzaldehyde emission in self-compatible *A. alpina* populations. In addition, the effects of benzaldehyde emission on the intensity of biotic interactions should be examined in field experiments.

### Floral scent composition differences between populations within self-compatible and self-incompatible groups

Variation in floral scent composition was structured differently within the categories of self-incompatible populations and self-compatible populations with high and low capacity for autonomous self-pollination. Several factors may contribute to the substantial variation in floral scent composition (Fig. 2B) and emission rate (Fig. 1C) among conspecific populations not differing in self-incompatibility or capacity for autonomous self-pollination. Geographical distance is likely not a major factor, since the correlation between geographical distance and scent composition dissimilarity was weak (Supplementary Data Fig. S4). Other possible factors include divergent selection on floral scent due to spatial variation in abundance and composition of assemblages of pollinators and herbivores (Gross *et al.*, 2016; Chapurlat *et al.*, 2019; Friberg *et al.*, 2019), population bottlenecks and genetic drift due to small population size (Delle-Vedove *et al.*, 2017), abiotic factors (Majetic *et al.*, 2009) and phylogeographic history (Soler *et al.*, 2011). For *A. alpina,* the relative importance of these factors likely varies among regions. The Scandinavian populations with a high capacity for autonomous self-pollination showed limited among-population variation but also low within-population variation, which correlates with the strongly reduced genetic diversity in these populations (Laenen *et al.*, 2018). By comparison, higher levels of genetic diversity, combined with divergent or relaxed selection on floral scent, could be responsible for the considerable variation among and within the French and Spanish populations with a low capacity for autonomous self-pollination. Similarly, divergent selection, historic factors and drift could contribute to differences between self-incompatible populations, which were characterized by high among-population variation and relatively low within-population variation in floral scent. Among the self-incompatible populations, the scent composition of the Greek populations differed clearly from that of the Italian populations (Fig. 2), but there was also considerable variation among Italian populations. The scent composition of two populations in the Apennines of central Italy (It2, It4) differed from that of the other populations (It5, It6, It7) in the Apennines despite occurring in close geographical proximity (40–80 km; Fig. 2). Most notably, the proportion (Supplementary Data Fig. S3), but not absolute emission (Fig. 1E), of benzaldehyde was substantially lower in the former populations. On the contrary, the two populations in the Apuan Alps (It8, It9), despite being genetically differentiated from populations in the Apennines (Ansell *et al.*, 2008), had a similar scent composition to the three latter Apennine populations (It5, It6, It7). This suggests that phylogeographic history and geographical distance are not major factors governing variation in floral scent among the Italian populations. However, additional studies are needed to disentangle the roles of phylogeography, divergent selection and random processes for among-population variation in floral scent traits in *A. alpina*.

### Conclusions

Our study demonstrates extensive intraspecific variation in floral scent in a widespread flowering plant. Scent emission rate was lower in self-compatible than in self-incompatible *A. alpina* populations. Surprisingly, however, the emission rate was not further reduced in self-compatible populations in Scandinavia with high capacity for autonomous self-pollination, compared with self-compatible populations in France and Spain with low capacity for autonomous self-pollination. Furthermore, differences in floral scent emission rate and composition between populations were considerable also within the groups of self-incompatible and self-compatible populations with high and low capacity for autonomous self-pollination. Our results therefore suggest that the evolution of floral scent is driven not only by the need for pollinator attraction, but is instead potentially the result of a complex set of factors including selection, population history and genetic drift. Future studies should examine how such factors interact to generate intra- and inter-specific variation in floral scent among flowering plants, and more closely investigate when, during transitions from self-incompatibility to self-compatibility and autonomous self-pollination, changes in floral signalling occur. Such information is crucial for predictions of how environmental change will affect selection regimes and evolutionary trajectories of natural populations.

## SUPPLEMENTARY DATA

Supplementary data are available online at https://academic.oup.com/aob and consist of the following. Figure S1: NMDS plot showing population centroids. Figure S2: out-of-bag probability for the Random Forest models. Figure S3: proportional benzaldehyde emission. Figure S4: relationship between geographical distance and scent composition dissimilarity. Table S1: identified floral scent compounds.

mcab007_suppl_Supplementary_S01

mcab007_suppl_Supplementary_S02
