# Supplementary material for: Evolution of floral scent in relation to self-incompatibility and capacity for autonomous self-pollination in the perennial herb Arabis alpina
Source: Ann Bot. 2021 Feb 8;127(6):737–47. doi: 10.1093/aob/mcab007 (PMC8103803; doi:10.1093/aob/mcab007)
Supplement: mcab007_suppl_Supplementary_S01 [file mcab007_suppl_supplementary_s01.docx]

**Supplementary Data. Figure S1-S4.**

**Evolution of floral scent in relation to self-incompatibility and capacity for autonomous self-pollination in the perennial herb *Arabis alpina***

Hampus Petrén, Per Toräng, Jon Ågren, Magne Friberg


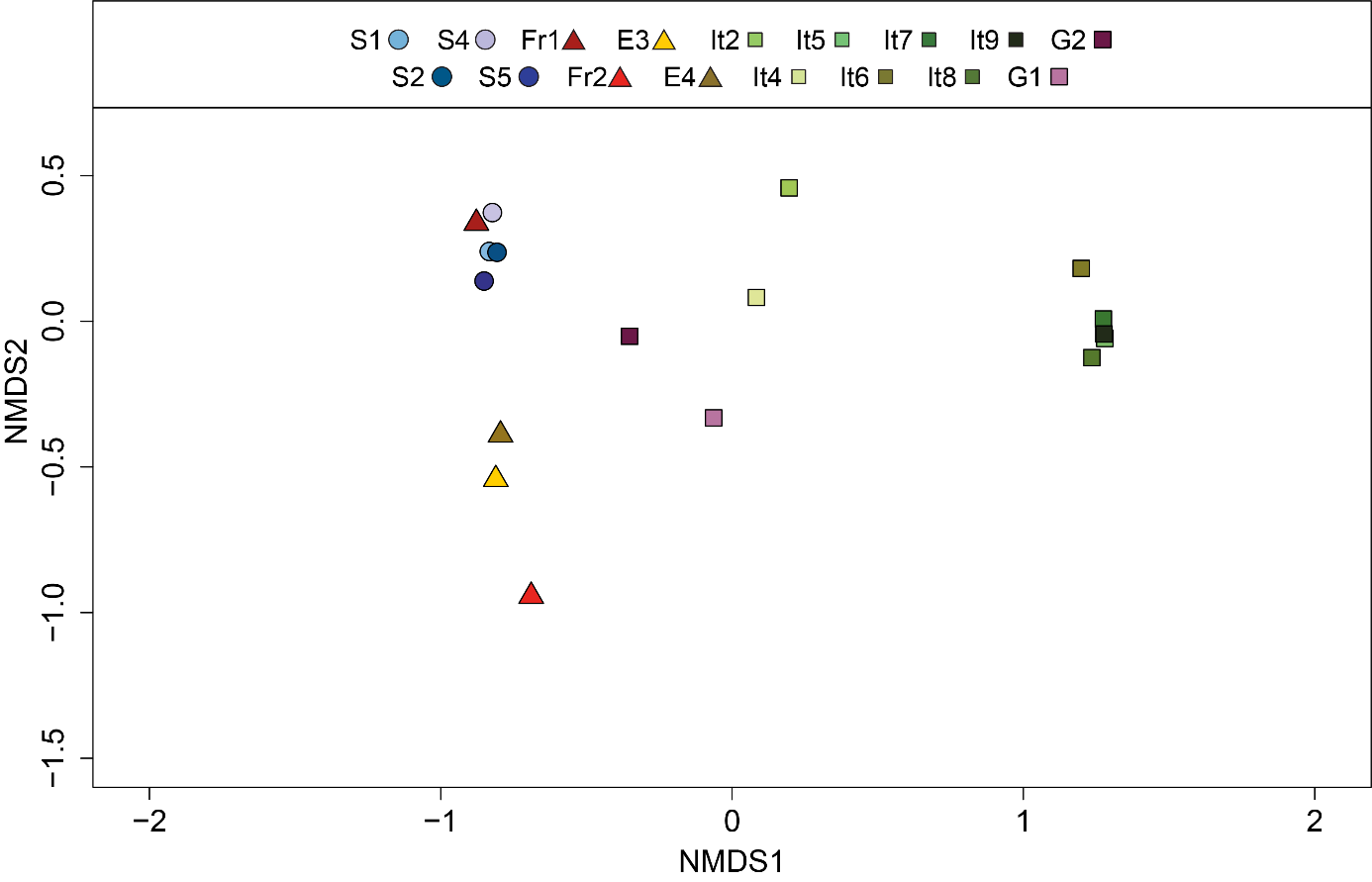


**Figure S1.** Non-metric multidimensional scaling (NMDS) plot showing only the population centroids for the samples in each population, for a clearer visualisation of the differences in floral scent composition between different *Arabis alpina* populations. Populations It5, It8 and It9 overlap in the rightmost cluster. See Table 1 for location of origin and characteristics of study populations.

**
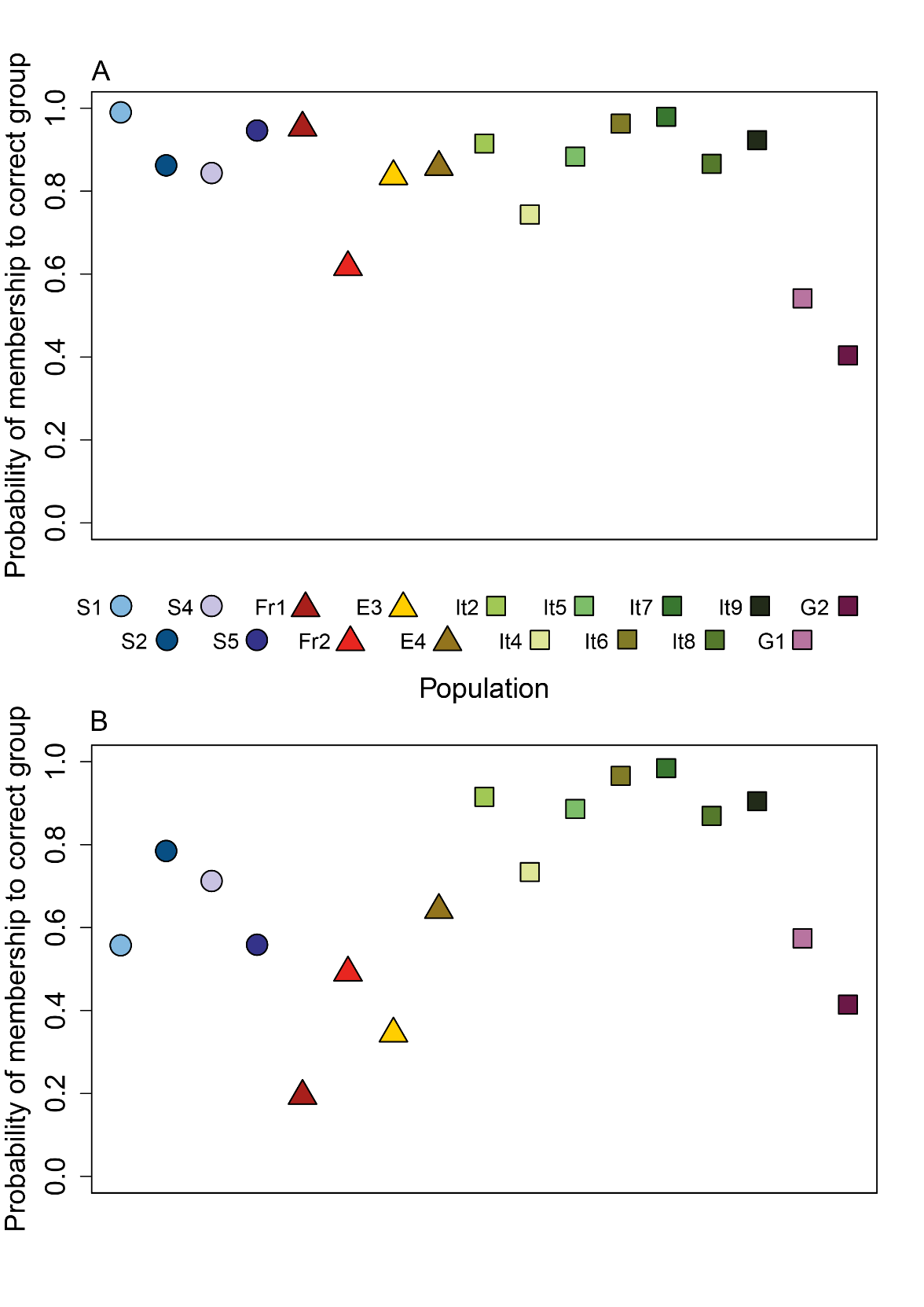
**

**Figure S2.** The “out of bag” (OOB) probability in the two Random Forests models of each *Arabis alpina* population belonging to the correct mating system category, based on population means of the relative proportions of scent compounds in each sample. (A) Model including two mating system categories (self-incompatible and self-compatible), (B) model including three mating system categories (self-incompatible, and self-compatible with low and with high capacity for autonomous self-pollination, respectively). See Table 1 for location of origin and characteristics of study populations.

**
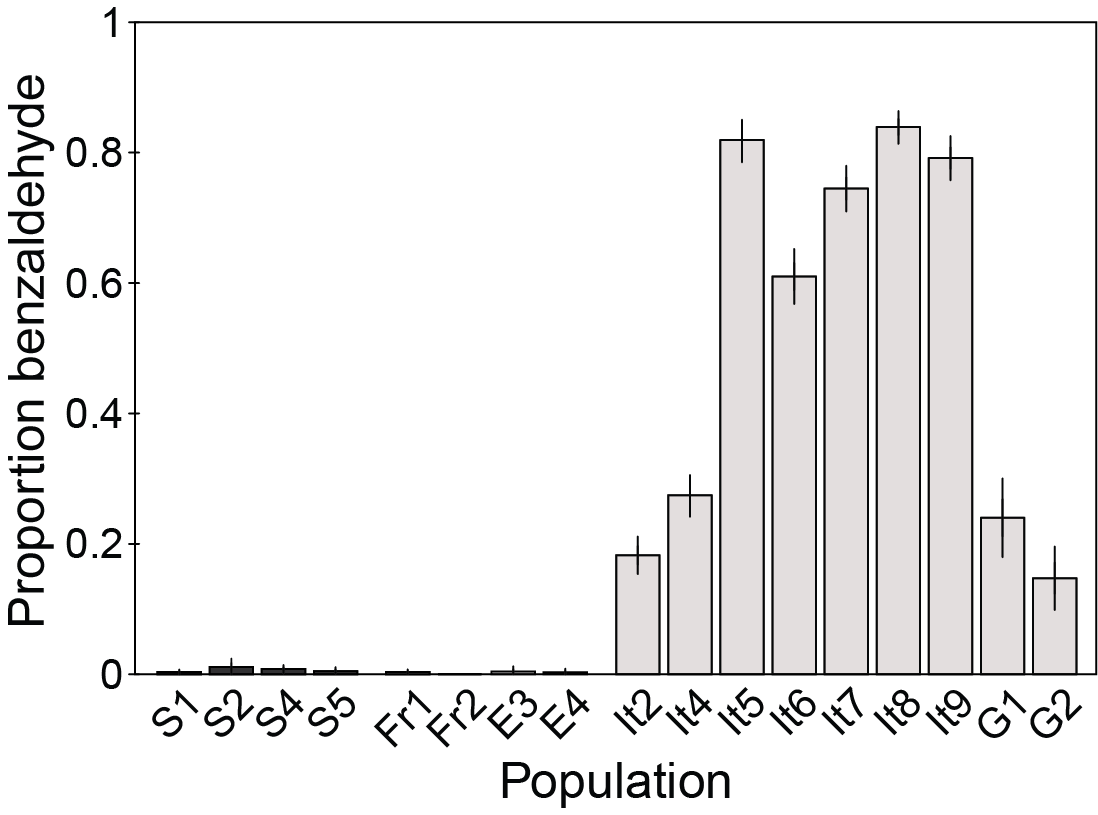
**

**Figure S3.** Benzaldehyde emission as a proportion of the total floral scent emission in the 17 study populations of *Arabis alpina*. Bars represent mean values, error bars represent 95% confidence intervals. Colours represent Scandinavian self-compatible populations with a high capacity of autonomous self-pollination (dark grey), French and Spanish self-compatible populations with a low capacity of autonomous self-pollination (medium grey) and self-incompatible Italian and Greek populations (light grey). S1, S2, S4, and S5, Scandinavian populations; Fr1 and Fr2, French populations; E3 and E4, Spanish populations; It2, It4, It5, It6, It7, It8, and It9, Italian populations; G1 and G2, Greek populations. See Table 1 for location of origin and characteristics of study populations.

**
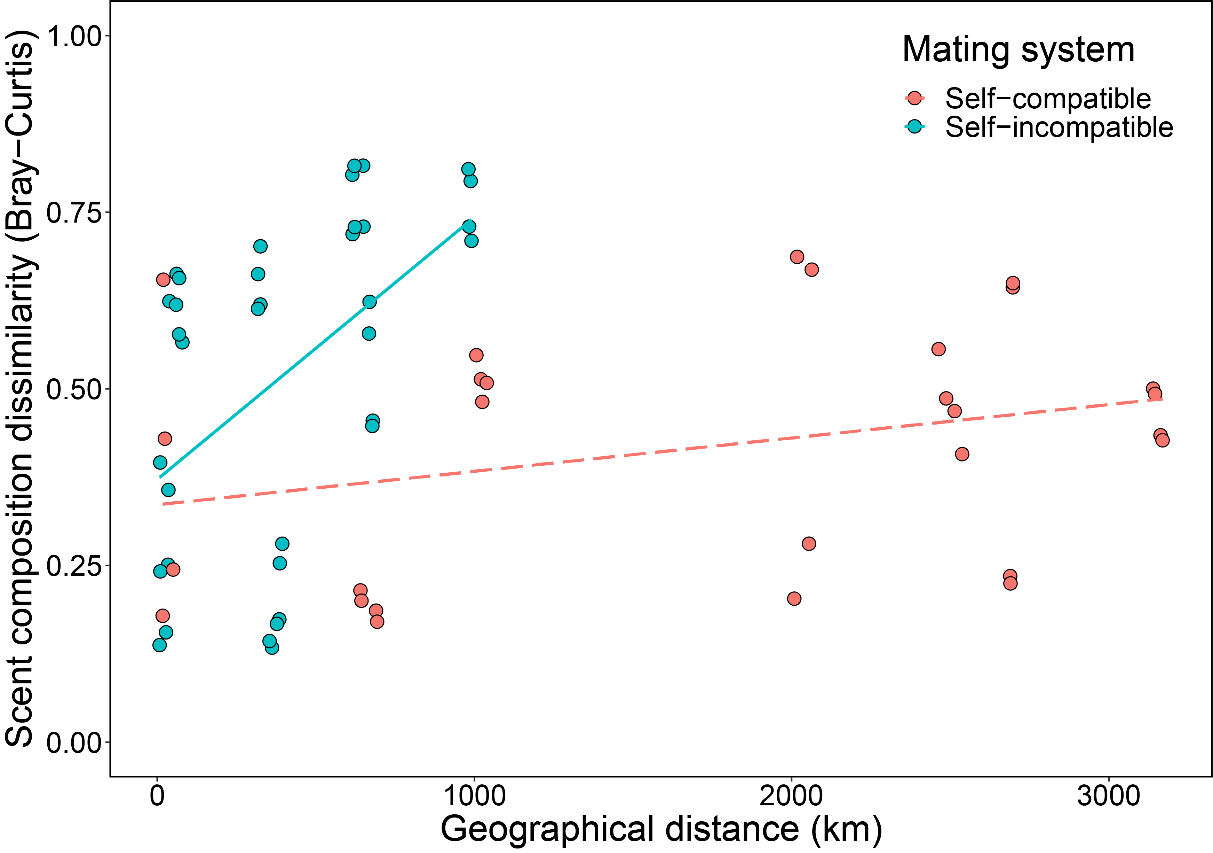
**

**Figure S4.** Relationship between geographical distance and scent composition dissimilarity for pairs of the eight self-compatible (red) and nine self-incompatible (blue) populations of *Arabis alpina*. Scent composition dissimilarity was calculated using average Bray-Curtis dissimilarities for all population pairs, where a higher dissimilarity indicates that populations differed more in floral scent. The correlation between geographical distance and scent composition dissimilarity was tested with Mantel tests within each of the two groups of populations, and is illustrated with the respective lines. A solid line indicates a statistically significant Mantel test (*P* < 0.05).
